# Editorial: Exploring the intersection of animal breeding, genetics, and genomics in modern agriculture

**DOI:** 10.3389/fgene.2026.1828383

**Published:** 2026-04-17

**Authors:** K. M. Davenport, Z. M. Estrada-Reyes, C. S. Wilson

**Affiliations:** 1 Washington State University, Pullman, WA, United States; 2 Prairie View A&M University, Prairie View, TX, United States; 3 USDA-ARS-Range Sheep Production Efficiency Research Unit, Dubois, ID, United States

**Keywords:** animal breeding and genetics, animal genomics, disease resilience, genetic diversity, reproductive efficiency

## Introduction

Modern agriculture relies on integrated farming approaches that combine advanced science, technology, and management practices to enhance efficiency, productivity, sustainability, and food quality while minimizing environmental impacts. This shift reflects a broader transition from traditional, labor-intensive methods to data-driven, technology-based, and systems-based production. For livestock and aquaculture systems, advances in animal breeding and genetics have substantially improved their productivity, health, and sustainability.

Over the past two decades, rapid progress in genomic technologies, such as whole-genome sequencing, transcriptomics, structural variant detection, gene editing, epigenetics, and high-throughput phenotyping, has greatly expanded our ability to dissect the genetic architecture of complex traits and implement precision breeding strategies. Despite these advances, translating genomic discoveries into tangible improvements in agricultural systems remains a key challenge.

This Research Topic, *Exploring the Intersection of Animal Breeding, Genetics, and Genomics in Modern Agriculture*, brings together 10 manuscripts spanning livestock, poultry, aquaculture, and camelid systems. Together, these contributions demonstrate how integrating genomics, transcriptomics, and economic analyses can accelerate genetic improvement, enhance disease resilience, and support sustainable livestock and aquaculture production under diverse environmental conditions.

## Genetic diversity

Maintaining genetic diversity is essential for long-term breeding progress, conservation of genetic resources, and resilience to environmental change. Zhu et al. conducted whole-genome resequencing of Chinese indigenous Kele pigs, revealing genetic diversity, a unique population structure, and selection signatures associated with economically important traits, such as fat deposition, muscle development, and immune function. These findings emphasize the value of indigenous breeds as reservoirs of adaptive genetic variation.

Two studies in this Research Topic focused on camel populations, which further highlighted the role of genomics in understanding adaptation to extreme environments. Ibrahim et al. characterized the genetic diversity and geographical structuring of Saudi Arabian dromedary camels, identifying gene sets associated with environmental adaptation and population isolation. Almathen and Salim provided a comprehensive review outlining the challenges and opportunities in camel genomics, particularly the need for standardized phenotyping and pedigree recording, improved genomic resources, and international collaboration to enable effective genomic selection.

One of the persistent questions in livestock improvement is whether the dissemination of superior germplasm translates into measurable economic benefits for producers. Gururaj et al. addressed this question through a causal impact assessment of Murrah buffalo germplasm adoption in Haryana, India. Using econometric approaches, their study provides evidence that progeny testing and the targeted dissemination of germplasm can significantly improve milk yield, reproductive performance, and the household income of farmers in this region. These findings support that breeding programs using genetics should be accompanied by extension services and proper reproductive management, including affordable artificial insemination, to fully realize the predicted economic gains.

## Disease resilience

Disease resilience remains an essential component of successful livestock production systems. Bernini et al. investigated the genomic relationships to bovine respiratory disease (BRD) using copy number variants and runs of homozygosity. The authors identified candidate genes and pathways involved in the immune response and lung development, highlighting the potential importance of structural variation in disease susceptibility. Their findings support the development of precision breeding strategies that integrate diverse genomic features, including structural variants, to improve animal health. Mir et al. investigated breed-specific immune responses to *Salmonella* Typhimurium in chickens, revealing differences in CXCLi1 expression and hematological profiles across breeds. Their work underscores the value of incorporating resistance traits from indigenous breeds into commercial populations, reinforcing the importance of conserving and leveraging genetic diversity to improve disease resilience.

## Efficiency of growth and reproduction

Reproductive efficiency and growth are critical drivers of productivity in livestock and poultry. Li et al. utilized whole-transcriptome sequencing across the hypothalamic-pituitary-ovarian axis in chickens, revealing complex regulatory networks involving lncRNAs, miRNAs, and mRNAs associated with egg-laying performance. By identifying key regulatory genes and pathways, this study provides valuable targets for marker-assisted and genomic selection. Similarly, Zhang et al. investigated miRNA profiles during sheep fat-tail development, identifying regulatory miRNAs involved in adipogenesis and lipid metabolism. These insights enhance our understanding of adaptive traits and fat deposition, both of which are economically significant in sheep production systems, particularly in harsh environments. Additionally, Bai et al. created a comprehensive transcriptome profiling dataset across 13 tissues in boars, providing a valuable resource for functional genomics and trait mapping in swine.

Expanding genomic tools to aquaculture and amphibian systems is critical as global demand for protein continues to rise. Zhang et al. developed sex-specific genetic markers in a species of frog, *Quasipaa spinosa*, which has been classified as a species at risk, providing a foundation for breeding strategies in frog aquaculture that promote greater growth. These strategies can substantially enhance production efficiency to meet increased protein demands worldwide and play a role in species conservation.

## Summary

Overall, the 10 studies in this Research Topic demonstrate the potential of integrating genomic technologies with phenotypic data and economic analyses to promote modern agricultural practices. From evidence of germplasm adoption benefits to molecular regulatory networks and population genomics related to disease and production traits, these contributions underscore the necessity of multidisciplinary approaches to modern breeding programs. We hope this Research Topic stimulates further research at the intersection of animal breeding, genetics, and genomics, and contributes to the development of livestock and aquaculture production systems that can meet the demands of a rapidly evolving global agricultural landscape.

